# Effectiveness of educational interventions for quality of life of parents and children with food allergy: A systematic review

**DOI:** 10.1097/MD.0000000000030404

**Published:** 2022-09-09

**Authors:** Jooyoung Cheon, Chung Min Cho, Hyo Jin Kim, Dong Hee Kim

**Affiliations:** a College of Nursing, Sungshin Women’s University, Dobong-ro 76ga-gil, Gangbuk-gu, Seoul, Republic of Korea; b College of Nursing, Seoul National University, 103, Daehak-ro, Jongno-gu, Seoul, Republic of Korea.

**Keywords:** child, education, effect, food allergy, parent

## Abstract

Educational intervention along with medical treatment is important to improve the quality of life of children with food allergies and their parents. This systematic review was designed to investigate the effects of education programs for parents and children with food allergies to provide a direction for developing an intervention program to improve their quality of life.

**Methods:**

A systematic review was conducted in accordance with the Preferred Reporting Items for Systematic Reviews and Meta-Analyses (PRISMA) guidelines. Relevant studies published between January 2010 and August 2021 were identified through a systematic search of 5 databases (PubMed, EMBASE, CINAHL, Cochrane Central Register of Controlled Trial, and Psycho Info).

**Results:**

A total of 2351 articles were identified. Of these, 9 met the inclusion criteria after duplicates were removed. Among these, only 2 studies, using the support and handbook provided, showed significant results on quality of life.

**Discussion:**

There is a lack of educational interventions for children with food allergies and their parents. Educational intervention, an essential intervention, can maximize medical treatment and improve overall quality of life. Hence, these interventions should be actively developed and applied in the future.

## 1. Introduction

Food allergy, an adverse reaction to a specific food antigen,^[1]^ is common worldwide and is becoming a major health issue.^[2,3]^ Its prevalence is higher in infants and young children than it is in adults.^[4,5]^ Food allergy can occur at various levels, such as skin, gastrointestinal, cardiovascular, respiratory, neurological, and systemic, and it can affect nutrition and growth development beginning at birth.^[6]^ Furthermore, children with food allergy are socially isolated and are at an increased risk of social-emotional and developmental difficulties. Food allergy also has an impact on school attendance^[7]^ and negatively affects children’s social activities, such as being bullied due to their allergies.^[8]^ Parents must prepare meals that limit exposure to the food on a daily basis. In addition, owing to concerns regarding the child’s nutritional status and health, they are burdened in various ways.^[9,10]^ These problems negatively affect quality of life for both children and their parents.^[7,9,11]^

To date, interventions for children and parents with food allergy have been developed and applied in various ways to solve their problems.^[12,13]^ There are immunotherapy and new pharmaceutical therapies for symptom relief and treatment. Furthermore, allergen avoidance therapy, the mainstay of management, is considered important.^[14]^ These interventions can be effective when applied in conjunction with educational interventions, such as peer or expert support, and education. Educational intervention can also be an effective strategy to improve quality of life for parents and children with food allergies by increasing their knowledge and self-efficacy for daily food allergy management and decreasing psychosocial problems.^[12]^

Therefore, in this study, a systematic literature review was conducted to analyze the research results on the effectiveness of educational interventions to improve the quality of life of children and parents with food allergies. The results presented an effective direction for the development of an intervention program to improve quality of life.

## 2. Methods

A systematic literature review was conducted to analyze the results of studies on the effects of education for parents and children with food allergies. This systematic review followed the Preferred Reporting Items for Systematic Reviews and Meta-Analyses (PRISMA) guidelines. Institutional review board permission is not required for conducting systematic review.

### 2.1. Search strategy

Databases were searched from inception and included PubMed, EMBASE, CINAHL, Cochrane Central Register of Controlled Trial, and Psycho Info databases from January 2010 to August 2021. The population, intervention, comparison, and outcome (PICO) for the systematic review were as follows. The study population consisted of parents and children with food allergies. Education programs were used as the intervention. A comparison was not performed. The outcome was quality of life. Keywords used for the literature review were food allergies, children, parents, and quality of life. For search terms, Medical Subject Heading (MeSH) terms were used in PubMed and Cochrane Library, and Emtree terms were used in EMBASE. In addition, related natural words were added, and Boolean operators (AND, OR, NOT) were combined between the search words to convert them into search expressions. Key words for researching food allergies included “Food hypersensitivity” [MesH], “Meals”[MeSH], “food allergy”[Emtree], and “meal”[Emtree]. For children, “Infant”[MeSH, Emtree], “Child, Preschool”[MeSH], “Child”[MeSH, Emtree], “preschool child”[Emtree], and “child hood” [Emtree] were selected as search terms. For Parents, “Parents”[MeSH, Entree], “Parents/education” [MeSH], “Parenting”[MeSH], “Child Rearing”[MeSH], “Parent-Child Relations”[MeSH], “Mother-Child Relations”[MeSH], and “Father-child Relations”[MeSH] were selected. Furthermore, patient education as “Topic”[MeSH], “parenting education”[Emtree], “child parent relation”[Emtree], “mother child relation”[Emtree], “father child relation”[Emtree], and “parent education”[Emtree] were also selected as search terms. For quality of life, “Quality of Life”[MeSH, Emtree] was selected. Articles were screened using the eligibility criteria, initially by title and then abstract, and finally by full text when necessary.

### 2.2. Eligibility criteria and exclusion criteria

Initially, we included all articles on the quality of life of parents and children diagnosed with food allergies. The articles to be analyzed were selected according to the following inclusion criteria.

1. An educational intervention study on the effects of food allergy prevention and physical and psychological management intervention. In this study, education referred to education being offered, information, or lectures by experts, counseling, training, and mentoring.
2. Experimental research
3. Quality of life as the outcome measure
4. Published Articles

Exclusion criteria were:

1. Intervention study on medication and immunotherapy
2. Only the abstract was available

### 2.3. Data extraction

The articles were downloaded by 2 researchers (DHK and JYC) using EndNote 20. They reviewed and screened the titles and abstracts and excluded those that did not involve an intervention. Furthermore, they independently examined the full text of the remaining articles using a standardized data extraction form to determine whether they met the inclusion criteria. Disagreements were resolved by discussion or, if necessary, by soliciting the opinion of a third researcher (JMC). Ultimately, all authors arrived at a consensus after a detailed examination of the articles.

### 2.4. Quality assessment

The methodological quality of the final selected articles was independently evaluated using Cochrane Risk of Bias 2.0 (RoB 2) for randomized trials and Risk of Bias In nonrandomized Studies of Interventions (ROBINS-I) for quasi-experimental research studies and single-group experimental studies. Each criterion for the risk of bias was judged by 2 reviewers and any disagreements were resolved by a third reviewer.

### 2.5. Data analysis and synthesis

A narrative synthesis of the data structured around the target and the type of intervention was undertaken.

## 3. Results

### 3.1. Results of the search

**Figure 1.**
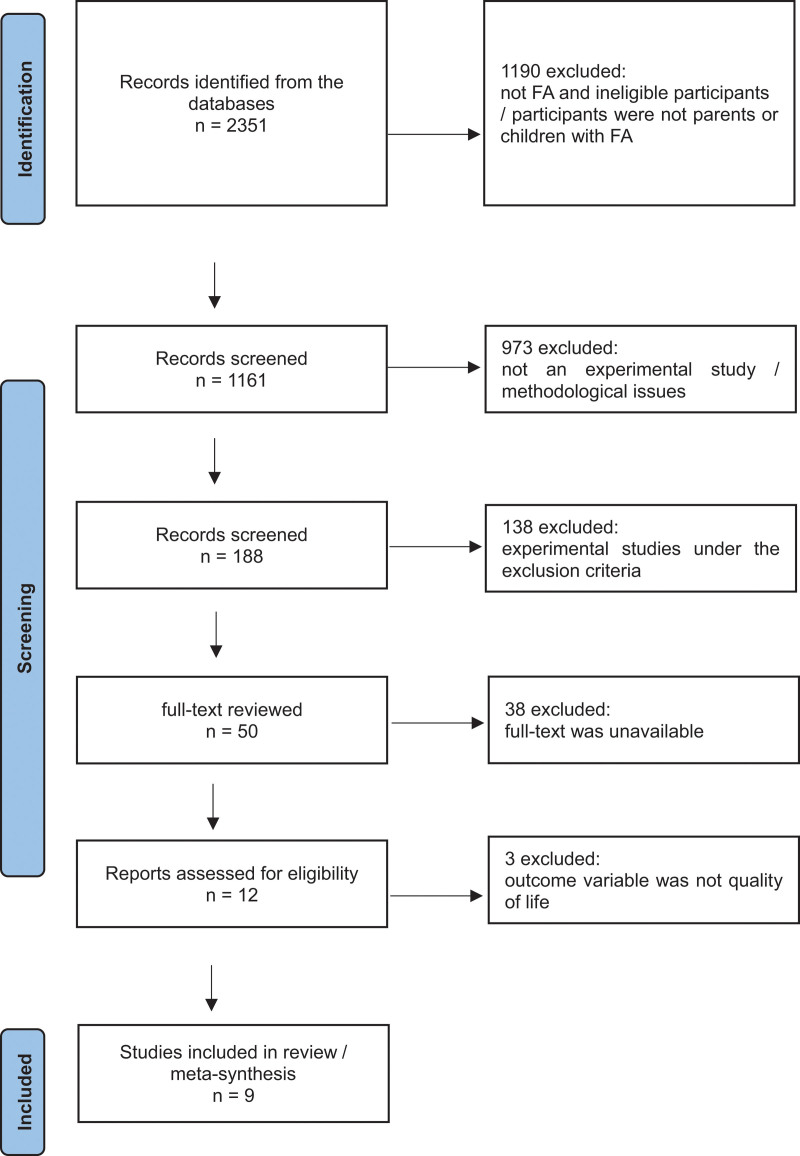
Flow chart of the identification of the relevant studies.

After duplicates were removed, a total of 2351 articles were retrieved. Among these, 2342 articles were removed after the titles and abstracts were reviewed based on the eligibility and exclusion criteria. Finally, 9 articles were selected and analyzed (Fig. 1).

### 3.2. Characteristics of included studies

The 9 included studies were published between 2016 and 2021. Of these, 4 were from North America,^[15–18]^ 3 from Australia,^[19–21]^ 1 from the UK,^[22]^ and 1 from Israel.^[23]^ Of these, 5 study designs were RCT,^[15–17,20,22]^ 2 were quasi-experimental studies,^[21,23]^ and 2 were on-group preposttest designs.^[18,19]^ The majority of the study subjects were parents (six cases),^[15,18–22]^ and 3 studies included children.^[16,17,23]^ The quality of life measurement tools used included the Food Allergy Quality of Life Questionnaire Child Form and Parent Form (FAQLQ-CF-PF),^[19,21–23]^ Food Allergy Quality of Life-Parental Burden Questionnaire (FAQL-PB),^[15,17,18,20]^ and Pediatric Quality of Life Inventory (PedsQL- adolescent version)^[16,19]^ (Table 1).

**Table 1:** Characteristics of studies included in the analysis (N = 9).

	First author (year)	Country	Study design	Sample size (Intervention/Control)	Participants	Child’s age (year) characteristics	QOL measurement	Follow-up period
1	Danchin^[19]^ (2016)	Australia	One group pre-posttest design	34	Parents	>7 History consistent with 1 of the 4 clinical scenarios; no history of confirmed diagnosis or management of food allergy by an allergist	FAQLQ The Pediatric Quality of Life Inventory (PedsQL)	Three months
2	LeBovidge^[15]^ (2016)	USA and Canada	RCT	153 (79/74)	Parents	0–18 Diagnosed FA within 1 year and had been prescribedan epinephrine autoinjector.	Food Allergy Quality of Life-Parental Burden Questionnaire (FAQL-PB).	Two months
3	Norman^[20]^ (2016)	Australia	RCT	75 (36/39)	Parents	2 to 16 Had confirmed or suspected nut allergy	FAQL-PB	Six months
4	Boyle^[22]^ (2017)	UK	RCT	200 (101/99)	Mothers	0–18 Diagnosed with food allergy, who were deemed to need an adrenaline auto-injector for the first time	Food Allergy Quality of Life Questionnaire (FAQLQ)	One year
5	Shemesh^[16]^ (2017)	USA	RCT	60 (30/30)	Parents and Children	13–17.5 Previously diagnosed with a food allergy and prescribed an autoinjector but never used it.	PedsQL—Adolescent version	One month
6	Weinberger^[17]^ (2019)	USA	RCT	60 (30/30)	Parents and children	9–17.5 With a peanut or tree nut allergy.	FAQL-PB	One month
7	Hiscock^[21]^ (2020)	Australia	Quasi-experimental design (pragmatic controlled trial)	373 (192/181)	Parents	0–12 Newly referred to the Allergy Clinic with suspected food allergy were eligible	FAQLQ	One year
8	Epstein-Rigbi^[23]^ (2021)	Israel	Quasi-experimental design	300 (88/212)	Parents and children	4–12 Who began OIT for IgE-mediated food allergy to milk, peanut, egg, sesame, or tree nuts	FAQLQ-Child Form (FAQLQ-CF) and Parent Form (FAQLQ-PF)	Not mentioned
9	Ramos^[18]^ (2021)	USA	One group pre-posttest design	Eight mentors and 10 mentees	Parents	(mentee) < 5 Diagnosed with FA within 1 year (mentor) 5–18 Diagnosed with FA at least 1 year prior	FAQL-PB	Six months

### 3.3. Intervention

The interventions provided were consultants,^[19,21]^ providing educational materials,^[15,20]^ cognitive behavioral therapy,^[22]^ practice,^[16,17]^ support,^[23]^ and peer mentoring.^[18]^ Among them, providing educational materials^[15]^ and support^[23]^ were reported as effective interventions for the children and parents’ quality of life (Table 2).

**Table 2:** Interventions (N = 9).

	Intervention	Intervention period	f/u	Effect on QOL	Statistical data
1^[19]^	**Consultation with a community-based general pediatrician** who completed an online Clinical Decision Support Training Program Provider: general pediatrician who completed an online Clinical Decision Support Training Program	Once or twice	-Baseline - 3 months	(N) There was no significant change in the child’s quality of life.	Not mentioned
2^[15]^	**The food allergy handbook** Components: allergen avoidance, symptom recognition and emergency treatment, practical strategies for managing allergies in daily life, coping with social and emotional challenges, educating others regarding food allergy, and teaching children regarding allergy management	Handbook group were instructed to read the handbook before the two-week follow-up.	- Baseline - Two weeks - Two months	(Y) At the 2-month follow-up, the handbook group had significantly greater improvement in quality-of-life scores than the control group.	Mean difference −0.48 (−0.79 to −0.16); *P* = .004
3^[20]^	**Booklet and monthly reminder text messages** Components: serving size guide and list of commercial foods containing specific nuts and recipes. Provider: dieticians	Six months text message: 5 times	Six months	(N) There was no significant change in FAQL-PB scores across the entire cohort at the 6 month follow-up.	Not mentioned
4^[22]^	**Cognitive behavioral therapy** Components: psychoeducation, including both the risk of living with high anxiety and the risk of fatal anaphylaxis for a child with food allergy, and coping thoughts and relaxation technique Provider: pediatrician in training, registered nurses, qualified psychologist	45 minutes, one session 15 minutes reinforcement (at 2 and 6 weeks)	-Baseline -Six weeks - 1 year	(N) There was no significant difference between the groups’ FAQLQ scores at either time-point.	At 6 weeks mean difference 0.13 (−0.26, 0.50; *P* = .54; d = 0.09) At 1 year mean difference 0.04 (−0.45, 0.54; *P* = .86; d = 0.03).
5^[16]^	**Practice self-injection** **(with an empty syringe**) Components: how to hold the syringe, put the point of the needle, insert, and dispose securely Provider: researchers	Not mentioned	-Baseline -One month	(N) There were no significant differences between group means.	*P* = .47
6^[17]^	**Touching their allergen and education** Components: hold a cup with a nut and touch the nut with their finger, education regarding being in proximity of and having contact with peanut and/or tree nuts Provider: physician	Time for each step was 5 minutes	-Baseline -One month	(N) There was no statistical difference between the groups.	*P* = .76
7^[21]^	**Consultation with a community-based general pediatrician** Provider: general pediatrician who had completed an online Clinical Decision Support Training Program	Not mentioned	-Six months -12 months	(Y)_ Food anxiety At 12 months, families reported less food allergy–related anxiety but more food-related social and dietary limitations compared with CC families.	Food anxiety mean difference − 0.29 (−0.50 to − 0.08); *P* = .01 Social and dietary limitations mean difference, 0.15 (0.12–0.19); *P* < .001
8^[23]^	**Supported by a medical clown (MC) during the induction week of oral immunotherapy (OIT) for food allergy** Provider: MC who graduated from the Dream Doctors organization The MC first met the patients at their arrival to the allergy clinic on the first day of OIT and supported them throughout the first up-dosing days	Four days (up to 6 hours/day).	One time	(Y)_children aged 8–12 years The support of a MC was most significantly associated with better QOL scores of children in allergen avoidance, dietary restrictions domains, and the total score. The support of a MC did not significantly impact children’s QOL (aged 4–12years), as perceived by their parents.	Allergen avoidance mean difference 1.33 (0.73–1.92); *P *< .001 Dietary restrictions mean difference 1.69 (1.12–2.26); *P *< .001 Total score mean difference 0.94 (0.44–1.44); *P *< .001 (*P* = .81)
9^[18]^	**Peer mentorship program** Mentor: primary caregivers of a child aged between 5 and 18 years, diagnosed with FA at least 1 year prior Communication: in-person meetings, phone, text, and email Components: tailored to the needs of the mentee.	Six months Frequency of contact was from 2 to 12 or more times.	Baseline Follow up: 6-month (unclear)	(N) Mentees’ scores decreased, but there were no statistically significant differences	Mentees’ scores decreased from 3.32 to 2.33 (95% CI = −2.32, 0.06) *P* = .06

### 3.4. Quality assessment

The quality appraisal results of the 9 studies are shown in Tables 3 and Table 4. As a result of evaluation using the ROB2 tool, 3 randomized controlled trials showed a low overall risk of bias.^[15,17,20]^ Of the remaining 2, 1 had some concern^[16]^ and the other had a high overall risk of bias.^[22]^ ROBINS-I found that 3 of the 4 showed serious^[18,19,21]^ overall risk of bias. Only 1^[18]^ with significant results showed a moderate overall risk of bias.

**Table 3:** Summary of the Cochrane risk of bias 2.0 (RoB 2; n = 5).

	First Author (year)	Randomization process	Deviations from the intended interventions	Missing outcome data	Measurement of the outcome	Selection of the reported result	Overall risk of bias
2	LeBovidge (2016)^[15]^	Low	Low	Low	Low	Low	Low
3	Norman (2016)^[20]^	Low	Low	Low	Low	Low	Low
4	Boyle (2017)^[22]^	High	Low	Low	Some concerns	Low	High
5	Shemesh (2017)^[16]^	Low	Low	Low	Low	Some concerns	Some concerns
6	Weinberger (2019)^[17]^	Low	Low	Low	Low	Low	Low

**Table 4:** Summary of the risk of bias in nonrandomized studies of interventions (ROBINS-I; n = 4).

	First Author (year)	Confounding	Selection of participants	Classification of interventions	Deviations from intended interventions	Missing data	Measurement of outcomes	Selection of the reported result	Overall risk of bias
1	Danchin (2016)^[19]^	Serious	Low	Low	Low	Low	Moderate	Low	Serious
7	Hiscock (2020)^[21]^	Serious	Low	Low	Low	Low	Moderate	Low	Serious
8	Epstein-Rigbi (2021)^[23]^	Moderate	Low	Low	Low	Low	Moderate	Low	Moderate
9	Ramos (2021)^[18]^	Serious	Low	Low	Moderate	Low	Moderate	Low	Serious

Only 2 studies^[15,23]^ that reported significant interventions on quality of life did not show a high or serious level of risk.

## 4. Discussion

Food allergies can have a profound impact on the overall quality of life of both children and their parents. This study aimed to develop an effective quality of life promotion program by analyzing the effects of various educational interventions.

The papers that were selected for analysis were published in the last 5 years (since 2016). Furthermore, only 9 studies were extracted, regardless of the effectiveness of the intervention, and only 2 studies were effective. Although studies on alleviating the symptoms of children with food allergies and their parents’ quality of life have been frequent, there have not been sufficient studies on the development and application of intervention programs that focus on quality of life. From this viewpoint, it was still too early to analyze the interventions in the included studies from various perspectives, such as the age at which the child was diagnosed, children’s age, and interventions. In addition, as a result of the quality assessment, high-risk studies were included, which suggested the need for delicacy in bias control for the development of accurate and effective interventions in future research designs.

The interventions used in the included studies were support, handbook provision, cognitive behavioral therapy, consultants, mentorship program, and practice. Among them, effective interventions included using support and handbooks provision.

Looking at the support intervention that worked, many studies and theories suggested that support had a positive effect on quality of life.^[24,25]^ Support plays a role in reducing anxiety, fear, and depression while enhancing positive emotions and facilitating recovery.^[26]^ Support for children and parents with food allergies can strengthen the existing healthcare delivery systems.^[27]^ The support analyzed in this study was that when children with food allergies came to the hospital, medical clowns were with them even for a short period of time. Various forms of support can be provided in the field of medicine. Therefore, it is necessary to develop cost-effective and sustainable support interventions to suit different situations.

The intervention that influenced parents’ quality of life was a handbook with educational contents, which included allergen avoidance, symptom recognition and emergency treatment, practical strategies for managing allergies in daily life, coping with social and emotional challenges, educating others about food allergies, and teaching children regarding allergy management. Patient and caregiver education has been proposed as a potentially useful adjunct to medical treatment to improve health-related quality of life. Educational interventions provide both patients and their caregivers with information regarding the condition and its treatment and can help them develop self-management skills. Education can also enhance quality of life for parents and caregivers by improving their self-management capabilities.^[28]^ In the future, the development of educational interventions considering various aspects is required because educational interventions require careful consideration of the learning content and the most effective process, including who is best to teach affected people and at what frequency and duration. Furthermore, its application in children should consider the child’s developmental stage.^[29]^

As mentioned earlier, the number and quality of the papers were insufficient for intervention analysis. Among the papers analyzed, insignificant interventions should be developed into more effective interventions by modifying them into interventions suitable for target children considering the timing, period, child’s age, hospital, home, and school-based perception. Therefore, various comprehensive attempts are required.

## 5. Conclusion

This study analyzed various educational interventions that could improve the quality of life of children with food allergies and their parents. Although there were many related papers that affected their quality of life, integrated and effective intervention development is still insufficient. The results of this study were not conclusive enough to make strong recommendations for future intervention. However, despite our limited results, we suggest that future interventions that use more methodologically sound designs to improve the quality of life should be actively and continuously conducted.

## Acknowledgments

This work was supported by the Basic Science Research Program through the National Research Foundation of Korea (NRF), funded by the Ministry of Education (NRF-2021R1A2C1006870).

## Author contributions

Conceptualization: Dong Hee Kim

Methodology: Jooyoung Cheon, Chung Min Cho, Dong Hee Kim

Validation: Jooyoung Cheon, Chung Min Cho

Formal analysis: Jooyoung Cheon, Chung Min Cho, Dong Hee Kim

Investigation: Hyo Jin Kim, Dong Hee Kim

Data curation: Jooyoung Cheon, Hyo Jin Kim, Dong Hee Kim

Writing—original draft preparation: Jooyoung Cheon, Hyo Jin Kim, Dong Hee Kim

Writing—review and editing: Jooyoung Cheon, Chung Min Cho, Dong Hee Kim

Supervision, Project administration, Funding acquisition: Dong Hee Kim
